# Inflammatory Lung Disease in Rett Syndrome

**DOI:** 10.1155/2014/560120

**Published:** 2014-03-17

**Authors:** Claudio De Felice, Marcello Rossi, Silvia Leoncini, Glauco Chisci, Cinzia Signorini, Giuseppina Lonetti, Laura Vannuccini, Donatella Spina, Alessandro Ginori, Ingrid Iacona, Alessio Cortelazzo, Alessandra Pecorelli, Giuseppe Valacchi, Lucia Ciccoli, Tommaso Pizzorusso, Joussef Hayek

**Affiliations:** ^1^Neonatal Intensive Care Unit, University Hospital Azienda Ospedaliera Universitaria Senese (AOUS), Viale M. Bracci 16, 53100 Siena, Italy; ^2^Respiratory Pathophysiology and Rehabilitation Unit, University Hospital, AOUS, Viale M. Bracci 16, 53100 Siena, Italy; ^3^Department of Molecular and Developmental Medicine, University of Siena, Via A. Moro 2, 53100 Siena, Italy; ^4^Child Neuropsychiatry Unit, University Hospital AOUS, Viale M. Bracci 16, 53100 Siena, Italy; ^5^Department of Maxillofacial Surgery, University of Siena, Viale M. Bracci 16, 53100 Siena, Italy; ^6^Institute of Neuroscience, CNR, Via G. Moruzzi 1, 56124 Pisa, Italy; ^7^Pathology Unit, University Hospital AOUS, Viale M. Bracci 16, 53100 Siena, Italy; ^8^Department of Medical Biotechnologies, University of Siena, Via A. Moro 2, 53100 Siena, Italy; ^9^Department of Life Sciences and Biotechnology, University of Ferrara, Via Borsari 46, 44100 Ferrara, Italy; ^10^Department of Neuroscience, Psychology, Drug Research and Child Health (Neurofarba), University of Florence, Area S. Salvi Pad. 26, 50135 Florence, Italy

## Abstract

Rett syndrome (RTT) is a pervasive neurodevelopmental disorder mainly linked to mutations in the gene encoding the methyl-CpG-binding protein 2 (MeCP2). Respiratory dysfunction, historically credited to brainstem immaturity, represents a major challenge in RTT. Our aim was to characterize the relationships between pulmonary gas exchange abnormality (GEA), upper airway obstruction, and redox status in patients with typical RTT (*n* = 228) and to examine lung histology in a *Mecp2*-null mouse model of the disease. GEA was detectable in ~80% (184/228) of patients versus ~18% of healthy controls, with “high” (39.8%) and “low” (34.8%) patterns dominating over “mixed” (19.6%) and “simple mismatch” (5.9%) types. Increased plasma levels of non-protein-bound iron (NPBI), F_2_-isoprostanes (F_2_-IsoPs), intraerythrocyte NPBI (IE-NPBI), and reduced and oxidized glutathione (i.e., GSH and GSSG) were evidenced in RTT with consequently decreased GSH/GSSG ratios. Apnea frequency/severity was positively correlated with IE-NPBI, F_2_-IsoPs, and GSSG and negatively with GSH/GSSG ratio. A diffuse inflammatory infiltrate of the terminal bronchioles and alveoli was evidenced in half of the examined *Mecp2*-mutant mice, well fitting with the radiological findings previously observed in RTT patients. Our findings indicate that GEA is a key feature of RTT and that terminal bronchioles are a likely major target of the disease.

## 1. Introduction

Rett syndrome (RTT), for a long time included among the Autism Spectrum Disorders (ASDs), is a nosologically distinct, genetically determined neurological entity associated in up to 95% of cases to* de novo* loss-of-function mutations in the X-chromosome-linked gene encoding the methyl-CpG-binding protein 2 (MeCP2) [1]. MeCP2, a ubiquitous protein particularly abundant in brain, is known to either activate or repress transcription [2, 3], is critical to the function of several types of cells (i.e., neurons and astroglial cells), and targets several genes essential for neuronal survival, dendritic growth, synaptogenesis, and activity dependent plasticity [4].

In its classical clinical presentation, RTT affects heterozygous females and shows a typical 4-stage neurological regression after 6 to 18 months of apparently normal development. RTT is a relatively rare disease, affecting about 1 : 10,000 female live births, although it represents the second most common cause of severe intellectual disability in the female gender [5, 6]. Preserved speech, early seizure, and congenital are well-known atypical variants often linked to mutations in genes other than* MECP2*, that is, the cyclin-dependent kinase-like 5 (*CDKL5*) in the early seizure variant and the forkhead boxG1 (*FOXG1*) in the congenital variant [6, 7].

Breathing disorders are considered a hallmark feature of RTT and represent a major clinical challenge [8]. To date, a large number of studies have been focusing on this particular characteristic of the disease, both in the clinical and experimental environments. Breathing abnormalities in RTT variably include/feature breath holdings, apneas, apneusis, hyperventilation, rapid shallow breathing, and spontaneous Valsalva maneuvers [9]. In particular, a highly irregular respiratory rhythm particularly during daytime is considered among the key symptoms of RTT [9–11]. Cumulating evidence indicates a predominantly hyperventilatory pattern with increased respiratory frequency and decreased expiratory duration, which is associated with frequent episodes of breath-holding/obstructive apnea or Valsalva breathing against closed airways during wakefulness [12–14]. However, the breath-holding/obstructive apnea phenotype of RTT is often confused in the related clinical literature with central apnea, which has fundamentally distinct neurological mechanisms [9, 15–26]. The wide spectrum of respiratory disorders detectable in RTT patients has been historically credited to brainstem immaturity and/or cardiorespiratory autonomic dysautonomia [9, 27, 28]. However, as the pathogenesis of the respiratory dysfunction in RTT appears far from being completely understood, alternative or complementary hypotheses can be formulated [29].

In particular, the potential role of oxidative stress (OS) mediators and the role of the lung itself in the pathogenesis of the respiratory dysfunction in the human disease are incompletely understood. More recently, biochemical evidence of redox imbalance and, in particular, enhanced lipid peroxidation, in blood samples from RTT patients, was further confirmed in primary skin fibroblasts cultures from patients [30–37], although the nature of the relationship, that is, whether causal or correlational, between* MECP2* gene mutation and abnormal redox homeostasis remains currently unclear. Significantly increased pulmonary alveolar-arterial gradient for O_2_, highly suggestive for an abnormal pulmonary gas exchange, has been previously described by our group in the majority of the examined RTT patients [29] and was found to be related in about a half of the cases to a pulmonary radiological picture partially mimicking that of the respiratory bronchiolitis-associated interstitial lung disease (RB-ILD) [38], one of the three lung conditions showing the stronger epidemiological association with tobacco smoke [39, 40].

However, to date, no information exists regarding the lung pathological lesions underlying the radiological changes observed in RTT patients.

Aims of the present study were to characterize the possible role of pulmonary gas exchange abnormality (GEA) in the pathogenesis of redox imbalance and respiratory dysfunction in RTT and to evaluate lung histology in an experimental mouse model of MeCP2 deficiency.

## 2. Methods

### 2.1. Subjects

In the present study, a total of *n* = 228 female patients with a clinical diagnosis of typical RTT and demonstrated* MECP2 *gene mutation were recruited (mean age, 12.9 ± 7.9 years; range, 1.5–32 years). RTT diagnosis and inclusion/exclusion criteria were based on a revised nomenclature consensus [6]. Clinical severity was assessed by the use of the clinical severity score (CSS), a specifically validated clinical rating system based on 13 individual ordinal categories measuring clinical features common in the disease [7]. Respiratory dysfunction on a clinical basis was categorized based on the corresponding Percy scale item (+ as minimal hyperventilation and/or apnea; ++ as intermittent hyperventilation and/or apnea; and +++ as hyperventilation and/or apnea with cyanosis) [41].

The corresponding *z*-scores for body weight, height, head circumference, and body mass index were calculated on the basis of validated RTT-specific growth charts [42]. Clinical stages distribution was: stage I (*n* = 4), stage II (*n* = 69), stage III (*n* = 92), and stage IV (*n* = 63). All the patients were admitted to the Rett Syndrome National Reference Centre of the University Hospital of the Azienda Ospedaliera Universitaria Senese. A total of 114 healthy and typically developed female subjects of comparable age (mean age, 12.9 ± 7.8 years; range, 1.6–32 years) were also enrolled in the study as a control population. Blood samplings from the control group were performed during routine health checks, sports, or blood donations obtained during the periodic checks. All the examined subjects were on a typical Mediterranean diet. The study was conducted with the approval by the Institutional Review Board and all informed consents were obtained from either the parents or the legal tutors of the enrolled patients.

### 2.2. Oxidative Stress (OS) Markers and Antioxidant Defence Evaluations

#### 2.2.1. Blood Sampling

Blood was collected in heparinized tubes and all manipulations were carried out within 2 h after sample collection. An aliquot (90 *μ*L) of each sample was used for reduced and oxidized glutathione assay. Blood samples were centrifuged at 2400 ×g for 15 min at 4°C; the platelet poor plasma was saved and the buffy coat was removed by aspiration. RBCs were washed twice with physiologic solution (150 mM NaCl). An aliquot of packed erythrocytes was resuspended in Ringer solution (125 mM NaCl, 5 mM KCl, 1 mM MgSO_4_, 32 mM N-2 hydroxyethylpiperazine-N-2-ethanesulfonic acid (HEPES), 5 mM glucose, and 1 mM CaCl_2_), pH 7.4 as a 50% (vol/vol) suspension for the determination of intraerythrocyte NPBI. Plasma was used for the NPBI assay.

#### 2.2.2. Intraerythrocyte and Plasma Non-Protein-Bound Iron (IE-NPBI)

Generally, NPBI is considered not only an OS marker but a prooxidant factor. In particular, IE-NPBI is a critical marker of hypoxia. IE-NPBI (nmol/mL erythrocyte suspension) was determined as a desferrioxamine- (DFO) iron complex (ferrioxamine) as previously reported [29] Plasma NPBI (nmol/ml) was determined as above reported for IE-NPBI [29].

#### 2.2.3. Plasma F_2_-Isoprostanes (F_2_-IsoPs)

F_2_-IsoPs, generated by free radical-catalyzed peroxidation of phospholipid-bound arachidonic acid, are considered specific and reliable OS markers* in vivo*. F_2_-IsoPs were determined by a gas chromatography/negative ion chemical ionization tandem mass spectrometry (GC/NICI-MS/MS) analysis after solid phase extraction and derivatization steps [43]. For F_2_-IsoPs the measured ions were the product ions at* m/z* 299 and* m/z* 303 derived from the [M−181]^−^ precursor ions (*m/z* 569 and* m/z* 573) produced from 15-F_2t_-IsoPs and PGF_2*α*_-d4, respectively [43].

#### 2.2.4. Blood Reduced and Oxidized Glutathione

Glutathione (*γ*-L-glutamyl-L-cysteinyl-glycine) is a tripeptide that plays an important role in protecting cells and tissues against OS [44]. Under nonoxidative and nitrosative stress conditions, over 98% of the glutathione is considered to be in the reduced form (GSH) [45], whereas under oxidative conditions GSH is converted to glutathione disulfide (GSSG), its oxidized form, with a resulting decrease in the GSH/GSSG ratio. As blood glutathione concentrations may reflect glutathione status in other less accessible tissues, measurement of both GSH and GSSG in blood has been considered essential as an index of whole-body glutathione status and a useful indicator of antioxidant defence [46]. Specifically, the GSH/GSSG ratio reflects the cellular redox status. Blood GSH and GSSG levels were determined by an enzymatic recycling procedure according to Tietze [47] and Baker et al. [48].

### 2.3. Cardiorespiratory Monitoring

In order to analyze the occurrence of apnoeas and hypopneas, breathing monitoring was carried out in RTT patients during wakefulness and sleep state by using portable polygraphic screening devices (SOMNOwatchTM plus, SOMNOmedics, Randersacker, Germany; importer for Italy Linde Medicale srl) for a mean recording time of 13 ± 0.5 h for each state. Monitoring included nasal airflow, arterial oxygen saturation by pulse oximetry, and respiratory efforts by abdominal and thoracic bands. Breathing patterns were analyzed for the presence of apnoeas and hypopnoeas according to the standardized definitions by the American Academy of Sleep Medicine [49] and the American Academy of Pediatrics [50]. Apnoeas were defined as a >90% airflow decrease for 10 sec, while hypopnoeas were defined as a >50% airflow reduction for ~10 sec associated with a decrease of 3% in oxygen saturation [49]. Apnoeas were categorized as obstructive (i.e., cessation of airflow for 10 sec with persistent respiratory effort), central (i.e., cessation of airflow for 10 sec with no respiratory effort), and mixed (an apnea that begins as a central apnea and ends up as an obstructive apnea). Apnoeas were further categorized as mild (10 to 15 sec), moderate (15 to 30 sec), and severe (>30 sec) on the basis of their recorded duration. The apnea-hypopnea index (AHI) was defined as the number of obstructive and central apnoeas and hypopnoeas per hour of sleep and calculated by dividing the total number of events by the total sleep time. An AHI > 15 during sleep was considered to be indicative of obstructive sleep apnea/hypopnea syndrome (OSAHS). All records were reviewed by a pneumologist with a longstanding expertise in OSAHS (i.e., coauthor M.R.).

### 2.4. Pulmonary Gas Exchange Analysis

**Figure 1:**
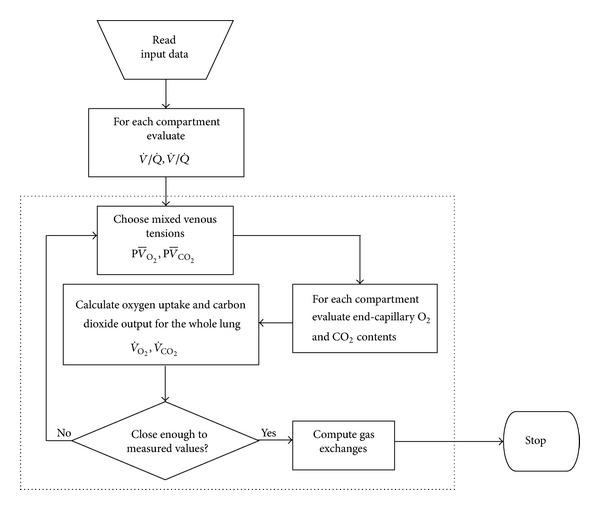
Algorithm for the noninvasive assessment of pulmonary gas exchange (Hanky Hapy gas analyzer version 1.2).

Pulmonary gas exchange was evaluated from direct measurements of total volume (*V*_tot_), respiratory rate, and expiratory fractions of CO_2_ and O_2_ by using a portable, commercially available gas analyzer (Hanky Hapy, version 1.2; Ambra Sistemi; Pianezza, Turin, Italy), as previously described [29]. The method to evaluate pulmonary gas exchange works essentially as a multicompartment model (Figure 1) and is essentially based on the classical West function [51]. Air gas sampling was obtained by applying a facial mask of appropriate size connected to the gas analyzer. Low invasivity and the easy-to-use features of the method allowed us to evaluate a relatively large population size of patients. Actually, the methodology does not require patient's cooperation and is therefore easily applicable to RTT patients and has been proven to be sufficiently simple, noninvasive, accurate, and precise in determining alveolar-arterial gradient lung exchange for O_2_ and ventilation/perfusion ratio (*V*/*Q*) inequalities. Respiratory rate, total ventilation, and expired gas composition were measured during either a 60-sec or 120-sec time period. *V*/*Q* distribution parameters were calculated by a minimizing mathematical function in order to reset to zero the differences between measured and calculated PaO_2_ and PaCO_2_. All respiratory measurements were carried out in duplicate, and the averages used for data analysis. Arterial blood for gas analyses was sampled from either the humeral or the radial artery, and PaO_2_, PaCO_2_, and pH values were determined using a commercially available blood gas analyzer (ABL520 Radiometer; Radiometer Medical A/S; Copenhagen, Denmark). Ventilation-perfusion (*V*/*Q*) inequalities (i.e., GEA) were classified as low, high, mixed, and simple mismatch. A “low” pattern indicates the presence of perfusion in poorly ventilated pulmonary areas; a “high” pattern points out the existence of high ventilation in poorly perfused pulmonary areas; a mixed pattern indicates a combination of the former two patterns; a simple “mismatch” was defined as a *V*/*Q* uncoupling showing a modest fraction of low *V*/*Q* ratios (1 to 0.1) and a modest fraction of high *V*/*Q* ratios (1 to 10). In order to account for the low PaCO_2_ values often encountered in RTT patients, standard PaO_2_ was calculated with the formula PaO_2_ = 1.66 × PaCO_2_ + PaO_2_ − 66.4, according to Sorbini et al. [52].

### 2.5. RTT Mouse Model: Murine Lung Histology

A total of (*n* = 4)* Mecp2* null mice and (*n* = 4) wild-type matched mice were examined. Experimental subjects were derived from heterozygous B6.129SF1-*Mecp2tm1Jae* knockout females (Mecp2+/−) [53]. Females were originally crossed to C57BL6/J for one generation, followed by breeding amongst offspring of the same generation with breeder changes, and were maintained on a mixed background. Mixed background reduced mortality and was necessary to obtain the high numbers of mice required by extensive analysis. Age-matched littermates were used in all experiments to control for possible effects of genetic background unrelated to the* Mecp2* mutation [54]. Mice were killed by decapitation at the thirty-eighth day of life; their lungs were removed rapidly and immediately frozen on liquid nitrogen. National and institutional guidelines were used for the care and use of animals, and approval for the experiments was obtained. Lungs were inflated with neutral buffered 10% formalin solution for about 24 h until adequate fixation. Each lung was dissected and sections were embedded in paraffin. Several 5 micrometres sections from each inclusion were stained with a standard hematoxylin and eosin staining protocol.

### 2.6. Statistical Data Analysis

All variables were tested for normal distribution (D'Agostino-Pearson test). Data were presented as means ± standard deviation or medians and interquartile range for normally distributed and non-Gaussian continuous variables, respectively. Differences between RTT and control groups were evaluated using independent-sample* t*-test (continuous normally distributed data), Mann-Whitney rank sum test (continuous nonnormally distributed data), chi-square statistics (categorical variables with minimum number of cases per cell ≥5) or Fisher's exact test (categorical variables with minimum number of cases per cell <5), one-way analysis of variance (ANOVA), Student-Newman-Keuls post hoc test, or Kruskal-Wallis test, as appropriate. Associations between variables were tested by either parametric (Pearson's coefficients) or nonparametric univariate (Spearman's rho) regression analysis. Predictive accuracy of apneas frequency/severity in identifying enhanced OS markers in RTT patients was calculated using a receiver operating characteristic curve (ROC) analysis, and an area under the curve value >0.5 was accepted to indicate good discrimination. The MedCalc version 12.1.4 statistical software package (MedCalc Software, Mariakerke, Belgium) was used for data analysis and a two-tailed *P* < 0.05 was considered to indicate statistical significance.

## 3. Results

### 3.1. Clinical Respiratory Dysfunction

Relevant demographic clinical characteristics for the examined RTT population are shown in Table 1. According to the specifically related items in the severity scoring system, all patients showed clinical signs for a respiratory dysfunction at different degrees, with moderate or severe dysfunction being detectable on a clinical basis in 81.6% (186/228) of the RTT patients.

### 3.2. Pulmonary Gas Exchanges

**Figure 2:**
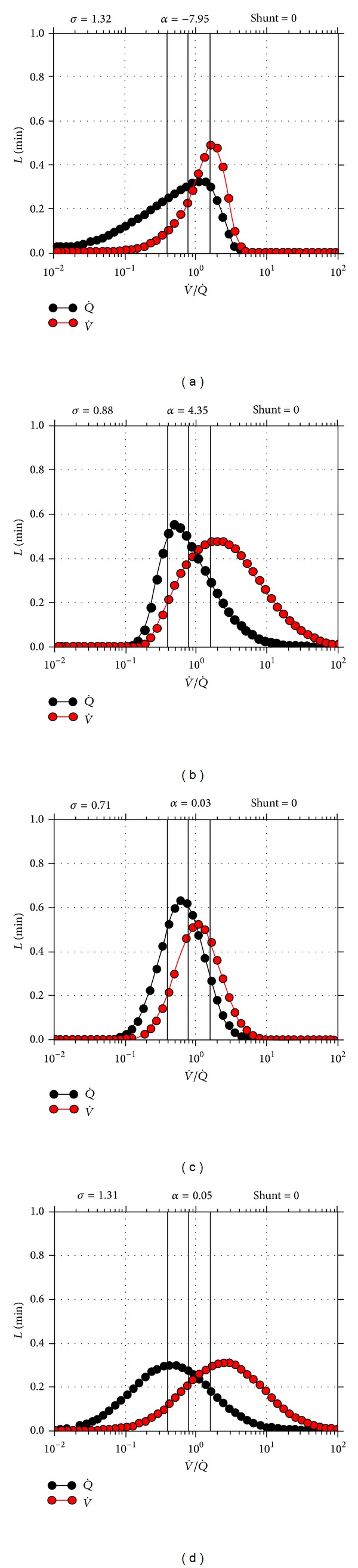
Representative pulmonary gas exchange abnormalities (GEA) patterns in patients with typical RTT and* MeCP2* gene mutation: (a) “low pattern” abnormality; (b) “high pattern” abnormality; (c) “simple *V*/*Q* mismatch”; and (d) “mixed pattern” abnormality. Ventilation-perfusion (*V*/*Q*) inequalities (i.e., GEA) were detectable in 80.7% of the whole RTT population, whereas only 19.3% of the patients showed a normal gas exchange. A “low” pattern (i.e., 34.8 of all GEA types in RTT) indicates the presence of perfusion in poorly ventilated pulmonary areas; a “high" pattern (i.e., 39.8% of all GEA types) points out the existence of high ventilation in poorly perfused pulmonary areas; a mixed pattern (i.e, 19.6% of all GEA types) is a combination of the former two patterns, while a “simple mismatch” (i.e., 5.9% of GEA types) is a *V*/*Q* uncoupling, showing a modest fraction of low *V*/*Q* ratios (1 to 0.1) and a modest fraction of high *V*/*Q* ratios (1 to 10).

Gas pulmonary exchange investigations demonstrated the existence of a variety of ventilation-perfusion inequalities (Figure 2 and Table 2) in more than 3/4 (i.e., 80.7%) of the whole RTT population; a “low” pattern (i.e., presence of perfusion in poorly ventilated pulmonary areas) was observed in 64 patients (28.1% of the examined whole RTT population), a “high” pattern (i.e., high ventilation in poorly perfused pulmonary areas) in 73 cases (32%), and a simple “mismatch” in 11 cases (4.8%), while a “mixed” pattern was present in 36 patients (15.8%). Overall, only 19.3% (44/228) of the RTT population showed a physiological (i.e., coupled *V*/*Q*) gas exchange pattern (RTT versus controls, chi-square: 138.472, DF = 4, *P* < 0.0001; chi-square for trend: 56.154, DF = 1, *P* < 0.0001).

Pulmonary gas exchanges parameters (Table 2) detected a general trend toward hyperventilation in the RTT patients, with mean total ventilation rates (*V*_tot_) of 6.3 ± 3.6 L/min (95% C.I. for the mean 5.8 to 6.7) versus 5.2 ± 2.1 L/min in the control subjects (*P* = 0.0028). Hyperventilation was absent in the “low” pattern of GEA, while being extreme in the “high” GEA pattern. Likewise, alveolar ventilation was the largest in the “high” pattern subpopulation of patients, with alveolar ventilation values usually >3 L/min for all GEA subcategories that is volumetrically consistent ventilation.

Blood gas analyses in RTT patients confirmed the presence of a relative hypoxia (PaO_2_: 87.5 ± 18.1 versus 98.7 ± 6.5 mmHg, difference ± SE: 11.2 ± 1.75, 95% C.I.: 7.76 to 14.6, *P* < 0.0001) and hypocapnia (PaCO_2_: 35.2 ± 7.5 versus 42.8 ± 5.8 mmHg, difference ± SE: 7.6 ± 0.08, 95% C.I.: 6.02–9.18, *P* < 0.0001), whereas blood pH was comparable between RTT and healthy controls (7.417 ± 0.043 versus 7.413 ± 0.045; difference ± SE: −0.004 ± 0.005854, 95% C.I.: −0.0138 to 0.005854, *P* = 0.4252). When hypocapnia was accounted for, standard PaO_2_ values in patients were found to be on average 17.6 ± 1.5% lower than those of their healthy control counterparts (PaO_2_: 81.1 ± 15.3 versus 98.7 ± 6.5, difference ± SE: 17.6 ± 1.5, 95% C.I.: 14.66 to 20.54, *P* < 0.0001) despite a normal-to-increased total volume (*V*_tot_) 5.8 ± 2.97 versus 5.2 ± 2.3 L/min, difference ± SE: −0.6 ± 0.317, 95% C.I.: −1.224 to 0.0239, *P* = 0.0594.

Remarkably, larger differences in hyperventilation were associated with consistently smaller intergroup differences (1-way ANOVA, *P* = 0.025) in PaO_2_ and even smaller differences when hypocapnia was accounted for (standard PaO_2_, *P* = 0.082), thus indicating a reduced efficiency of pulmonary exchange despite normal pH values. However, the physiological dead space, as calculated by the Bohr equation, was found to be at the upper physiological limits (i.e., 30 to 45% of the *V*_*t*_ in the healthy control population) in the “no mismatch,” “simple mismatch,” and “low” patterns (41.2 to 45.0 *V*_*t*_%), whereas it appears to be increased up to 55.4 ± 10.8 *V*_*t*_% and 54.7 ± 15.5 *V*_*t*_% in “high” and “mixed” patterns, respectively. These findings confirm the occurrence of a reduced efficiency of pulmonary exchanges in the RTT population, with a statistically significant relationship between respiratory rate and Bohr's physiological dead space (rho = 0.144, *P* = 0.0303). Overall, oxygen uptake (*V*_O_2__) and carbon dioxide production (*V*_CO_2__) values appear to be lower than those of healthy controls subjects (*V*_O_2__: 250 to 300 mL/min and *V*_CO_2__: 200 to 250 mL/min, resp.). Likewise, respiratory exchange ratios (i.e., *V*_CO_2__/*V*_O_2__) in the RTT patients were accordingly higher than those observed in healthy controls (1.56 ± 1.23 versus 0.81 ± 0.32, *P* < 0.0001).

### 3.3. Redox and Antioxidant Status

The results of the redox and antioxidant markers in RTT patients showed significantly increased plasma levels of non-protein-bound iron (NPBI) (~2-fold), F_2_-isoprostanes (F_2_-IsoPs) (~2.9-fold), reduced glutathione (GSH) (~1.4-fold), oxidized glutathione (GSSG) (~50-fold), and intraerythrocyte NPBI (IE-NPBI) (~1.5-fold) as compared to healthy control subjects (Table 3). Consequently, a significantly decreased GSH/GSSG ratio (~−15-fold) in patients was evidenced.

### 3.4. Cardiorespiratory Monitoring

Cardiorespiratory monitoring showed a significant prevalence of obstructive apnoeas both during the sleep and the wakefulness states in RTT patients, with median rates of obstructive apnoeas of 17.7/h and 6.2/h, respectively (Table 4). Of note, obstructive episodes were more prevalent as compared to central events by 25.3- and 15.5-fold during the wakefulness and sleep state, respectively. The lowest recorded SpO2 values during the apnoeic events were 78.8 ± 13.1%. Apneas during the sleep phase were detectable in 63.6% (145/228) of patients, with a mean AHI of 15.9 ± 4.69. Positive criteria for OSAHS (AHI > 15) were present in 27.2% (62/228) of the whole RTT patients population.

### 3.5. Relationship between Redox Imbalance and Apnea Frequency/Severity

Statistically significant positive correlations were observed between recording of apneas, independently of the degree of severity, and IE-NPBI (rho coefficients, range: 0.324 to 0.358;* P* values, range: 0.0024 to 0.0089) or GSSG (rho coefficients range: 0.258 to 0.267; *P* values, range: 0.0392 to 0.0156) (Table 5). On the other hand, positive relationships between apneas and p-NPBI (rho: 0.265, *P* = 0.0346) or F_2_-IsoPs (rho: 0.305, *P* = 0.0142) were also observed but limited to the most severe events only. A significant inverse relationship between moderate apneas and GSH to GSSG ratio was present (rho: −0.247, *P* = 0.0488). An average number of >7.4/h for total apneas, >0.8/h for moderate apneas, and >2 for severe apneas (either recorded during wakefulness or sleep states) were found to be predictive for increased IE-NPBI plasma levels in patients, with 50% to 80.7% sensitivity, 55.3% to 82.02% specificity, 55.3% to 65% positive predictive value, and 62.7% to 80.8% negative predictive value (*P* values for the AUC, range: 0.0044 to 0.0163) (Table 6). In contrast, frequency/severity of the recorded apneas was not predictive for plasma levels of F_2_-IsoPs in RTT patients (*P* values for the AUCs, range: 0.3149 to 0.9487).

### 3.6. Relationship between Redox Imbalance and Pulmonary Gas Exchange Abnormality

When evaluated as a function of GEA in RTT, striking differences in the redox/antioxidant markers levels were detectable among the different categories of pulmonary *V*/*Q* inequality, ranging from values comparable to those of the control group in the “no mismatch” group to significant redox/antioxidant imbalance in the various GEA patterns (Table 7). In particular, F_2_-IsoPs plasma levels were approximately proportional to the degree of severity for *V*/*Q* abnormality, with increase of ~1.7-fold for “simple mismatch,” ~2.4-fold for “low” patterns, ~2.8-fold for “high” patterns, and ~3.7-fold for “mixed” patterns, as compared to patients without detectable GEA.

### 3.7. Mutant* Mecp2* Murine Lung Histology

**Figure 3:**
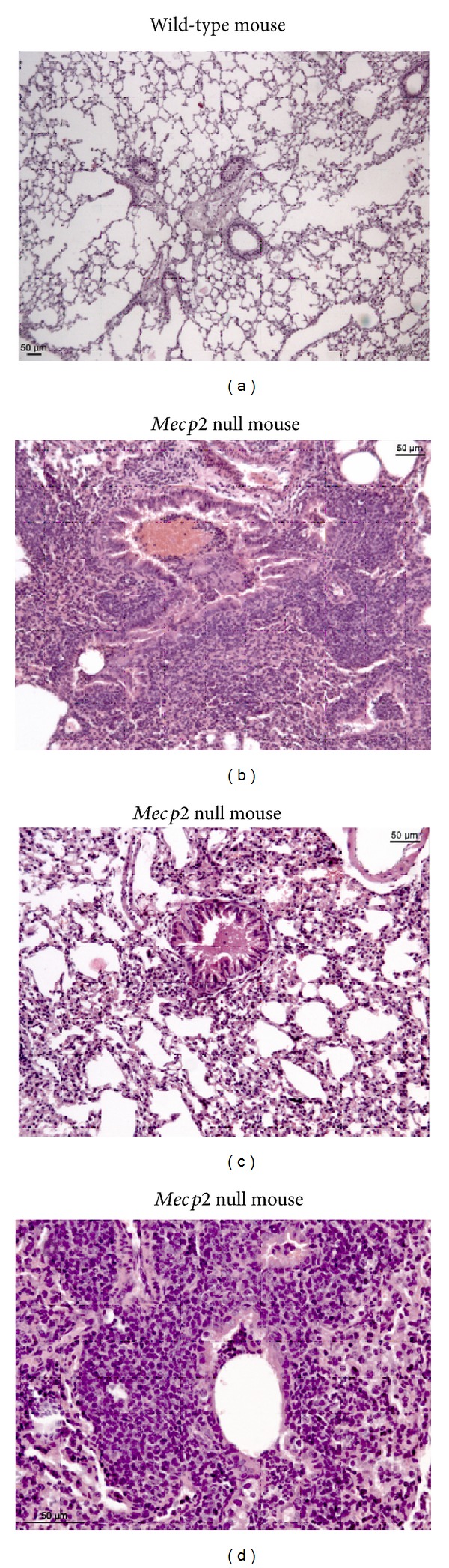
Lymphocytic bronchiolitis and desquamative alveolitis in* Mecp2 *null mice. (a) Wild-type mouse lung: normal histological features (magnification 25x), (b)* Mecp2* null mouse: peribronchiolar lymphocyticinfiltrate (magnification 50x), (c)* Mecp2* null mouse lung: desquamative alveolitis with mild amount of alveolar exudate (50x), and (d)* Mecp2* null mouse lung: terminal bronchiolitis at higher magnification (200x) lymphocytes and histiocytes infiltrates outside and inside thickened terminal bronchioles, with a resulting picture of lymphocytic bronchiolitis.

The results of lung histology in the* Mecp2* null RTT mouse models showed a picture of nonspecific lymphocytic bronchiolitis associated with lymphocytic vasculitis (Figures 3(b) and 3(d)) and desquamative alveolitis in a half of the examined mutant mice, whereas no significant histological abnormalities were observed in the wt animals.

## 4. Discussion 

Respiratory dysfunction in RTT appears to be far more complex than previously thought. Specifically, the findings of the present study indicate that, besides brainstem dysfunction, several intertwined critical factors, either directly or indirectly related to the disease, appear to concur to adversely affect respiratory function in RTT patients. In particular, our findings strongly support the hypothesis that the respiratory behavior in RTT, historically credited to neurological dysfunction, can be considered as the result of a previously unrecognized inflammatory process and/or abnormal immune response [55].

Abnormal pulmonary gas exchange, as the result of the imbalance between *V* and *Q*, is a main cause of hypoxemia [56]. While confirming the coexistence of a relative hypoxia (−17.6 ± 1.5% as compared to a cohort of healthy controls; 95% C.I.: −14.66 to −20.54) and abnormal pulmonary gas exchange in over 3/4 of the patients with typical RTT, we identified for the first time the relative distribution for the different GEA patterns. GEA was detectable in ~80% of the RTT patients, with “high” and “low” patterns dominating over “mixed” and “simple mismatch” types of pulmonary *V*/*Q* inequalities. This *V*/*Q* behavior here observed could be linked to either the presence of unventilated pulmonary areas in the low patterns (i.e., unrecognized pulmonary dysventilation or microatelectasis) or unperfused areas in the high patterns (i.e., unrecognized pulmonary microembolism). Therefore, our findings strongly suggest that GEA is a key feature of respiratory dysfunction in RTT that brainstem immaturity [9, 12] and/or cardiorespiratory autonomic dysautonomia [13, 14], both repeatedly evoked in the disease, are* per se* evidently unable to explain. It is interesting to observe that several features of the respiratory behaviour observed in RTT patients, chiefly hyperventilation, can be interpreted as compensatory mechanisms rather than the effects of dysfunctional brainstem activity and/or cardiorespiratory autonomic dysautonomia. However, hyperventilation, as a likely compensatory mechanism in order to overcome the negative effects of GEA, can lead to an adverse increase of the physiological dead space, as thus reducing the fraction of ventilation that effectively participates in alveolar gas exchanges. In addition, hyperventilation can lead to severe hypocapnia, a very frequent finding in RTT, thus further decreasing the effective (i.e., normalized to PaCO_2_) PaO_2_ levels. The observed decrease in *V*_O_2__ and *V*_CO_2__ could also be potentially regarded as possible effects of compensatory mechanisms, although more investigation is needed to understand this unexpected feature of the RTT lung pathophysiology.

OSAHS is described as repetitive obstructions of the upper airways during sleep, causing concomitant episodes of systemic hypoxia and associated cardiovascular and metabolic pathologies, with an estimated prevalence of 0.7% to 1.8% in the general paediatric population [57–62] and of 2% for adult women and 4% for adult men [63–65]. The condition can be difficult to diagnose clinically, although even mild-to-moderate obstructive sleep apnoea can result in adverse neurobehavioral consequences and negatively affect quality of life [58, 59]. Our cardiorespiratory monitoring data in a relatively large cohort of RTT patients indicate a significantly increased prevalence of OSAHS in this patient population (i.e., 27.2%) and confirm the presence of clinically significant apneas also during wakefulness. These findings indicate that RTT should be added to the list of the already known heterogeneous pediatric conditions carrying an increased risk for OSAHS, including Down's syndrome, neuromuscular disease, craniofacial abnormalities, achondroplasia, mucopolysaccharidoses, and Prader-Willi syndrome. Although the overwhelming majority of previous polysomnographic studies in RTT patients have reported a prevalence of apnoeas of central origin [15–26], our findings indicate that obstructive apnoeas are far more common in typical RTT than previously reported.

Human and experimental evidence indicate that OSAHS and intermittent hypoxia can be associated with enhanced OS, although conflicting reports exist [66–76]. In the present study, we confirm the coexistence of a significant redox abnormality in RTT patients. However, the relationship between upper airways obstruction/intermittent hypoxia and OS status in RTT appears to be limited to the generation of a prooxidant status, as indicated by the link here observed between IE-NPBI, but not F_2_-IsoPs, and apneas. Iron is a major player in redox reactions, as it has been known for a long time that redox-active iron is one of the most active sources of OS [77]. Furthermore, iron release is much higher under hypoxic conditions than under normoxia [78, 79], so that hypoxia paradoxically represents a condition of OS that is consistent with a condition of ischemia-reperfusion injury. Released iron can diffuse out of the erythrocytes and the diffusion is higher with hypoxic erythrocytes [79]. Intermittent hypoxia can affect the stability of the bond of iron to the tetrapyrrole ring of protoporphyrin, thus releasing iron inside the erythrocytes, along with hemoglobin autoxidation [80]. However, it becomes clear that mechanisms other than intermittent hypoxia should be the major sources of enhanced OS in RTT. Actually, in the present study, we observed a very intimate relationship between redox abnormality and GEA in RTT. These results suggest that chronic, rather than intermittent, hypoxia resulting from a pulmonary *V*/*Q* inequality is likely the main source of systemic OS in RTT. To this regard, it is of relevance that we detected a dramatic increase of GSSG in RTT patients, with a parallel dramatic decrease of the GSH/GSSG ratio, thus strongly suggesting the coexistence of a chronic OS status.

While future studies are obviously needed to address this major topic, several other factors apparently concur to adversely affect the respiratory function and, either directly or indirectly, contribute to pulmonary gas exchanges impairment in RTT patients. These factors likely include abnormal erythrocyte shape with oxidative membrane damage [81], microvascular dysplasia [82], alterations in the vascular/endothelial system [83], mitochondrial dysfunction [84–87], subclinical biventricular myocardial dysfunction [88], systemic oxidative stress [29, 35], and, chiefly, subclinical inflammatory processes [38, 55].

Over the last decade, several experimental animal models have been developed in which the* Mecp2* allele has been modified to prevent production of a fully functional Mecp2 protein. In particular, several experimental mouse models of Mecp2 deficiency have been established in mice, ranging from null-*Mecp2 *mutations to specific point mutations mimicking those observed in humans, phenocopying several motor and cognitive features of RTT patients. In particular, the irregular breathing pattern observed in human RTT has been replicated in several mutant mouse models to varying degrees of fidelity, although the corresponding respiratory phenotype varies among different mouse strains [89–91], with mutations in the* Mecp2* gene leading to disparate respiratory phenotypes. For instance, in* Mecp2*tm1.1Jae null (hemizygous) mice on a mixed-strain background [92], the principal phenotype is tachypnea along with hyperventilation similar to human RTT [93], whereas in* Mecp2*tm1.1Bird null or heterozygous mice on a pure C57BL/6J background [94] the principal phenotype is repetitive spontaneous central apnea [95–97], whereas the* Mecp2*tm1.1Bird male mice provide an excellent animal model of spontaneous central apnea and possibly obstructive apnea [28]. A more recent study indicates that a clinically relevant RTT endophenotype, that is, tachypnea with a shortened expiratory time, appears to be more faithfully reproduced in Mecp2tm1.1Jae female mice [98]. Despite the fact that* MeCp2* mutant mouse models cannot model all aspects of the human RTT, certainly they do recapitulate many aspects of the disease and are generally accepted as excellent tools to study MeCP2 function. In this context, our findings of a lymphocytic bronchiolitis in a half of the examined* Mecp2*-null mice are highly suggestive of a previously unrecognized inflammatory lung disease in RTT patients and are well fitting with our prior observations of radiological features at high-resolution computed tomography partially overlapping with those of RB-ILD [38] and including micronodules (i.e., inflammatory infiltrates in the smaller airways, such as terminal bronchioles and/or alveoli), “ground glass opacities” (i.e., radiological signs of alveolar inflammation), and, remarkably, thickening of bronchiolar walls (a radiological sign of inflammatory infiltrates in the terminal bronchioles). Further studies are obviously needed in different* Mecp2*-mutant animal models in order to ascertain prevalence and possible differences related to different mouse strains.

Cumulating evidence indicates that RTT is a multisystemic disease, which, besides the brain, is known to affect several organs and systems, including the autonomic nervous system [12–14], microvascular/endothelial system [12, 13], bone [99], heart [88, 100], red blood cells [81], the gastrointestinal tract [101], and the immune system [102, 103]. Our study strongly supports the concept that the lung is a previously unrecognized major target organ in this genetically determined neurodevelopmental disease and that pulmonary GEA is likely a key feature within the multisystemic characteristics of the disease.

## 5. Conclusions

The findings of the present clinical study confirm the emerging concept according to which no single putative mechanism can account for all the complexity of the respiratory behaviour exhibited by RTT patients. The present study indicates that (1) pulmonary GEA, not brainstem immaturity, is likely the key feature of respiratory dysfunction in RTT; (2) the RTT-related GEA is likely the result of several contributing factors, involving OS and chronic subclinical inflammation; and (3) terminal bronchioles and alveoli are likely a major, under-recognized, inflammatory target of the disease. Moreover, these data confirm the clinical relevance of respiratory dysfunction in this rare neurological disease as a valuable pathophysiological model for a better understanding of the complex involvement of the lung in a multisystemic disease. Our findings strongly support the hypothesis that the respiratory behavior in RTT, historically credited to neurological dysfunction, is rather the result of an inflammatory process and/or abnormal immune response.

## Figures and Tables

**Table 1:** Relevant demographic and clinical characteristics of female subjects with Rett syndrome.

Variables	
Patients (*N*)	228
Age (years)	12.9 ± 7.9^§^
Body weight (RTT *z*-score for age)^1^	0.025 ± 1.12
Body height (RTT *z*-score for age)^1^	−0.05 ± 1.13
Head circumference (RTT *z*-score)^1^	−0.22 ± 1.11
Body mass index (BMI) (RTT *z*-score for age)^1^	−0.36 ± 1.45
Clinical severity score (CSS)^2^	17.4 ± 7.3
Tachypnea^a^	59 (25.9 %)
Respiratory dysfunction on a clinical basis^b^	
+	42 (18.4 %)
++	127 (55.7 %)
+++	59 (25.9 %)
Additional clinical features	
Air-S^c^	64 (28.1 %)
Severe GERD^d^	27 (11.8 %)

^1^Calculated *z*-scores for age are referred to a validated Rett syndrome-specific growth charts [42].^ 2^Clinical severity score was defined according to Neul et al., 2008 [7]. ^a^Tachypnea was defined as a respiratory rate >1.8 times (i.e., above the upper quartile) of the expected respiratory rate for age and gender; ^b^respiratory dysfunction was categorized based on the corresponding Percy's clinical severity scale item [41]; ^§^mean  ± SD; ^c^Air-S: abnormal air swallowing; ^d^GERD: gastroesophageal reflux disease.

**Table 2:** Pulmonary gas exchange abnormality (GEA) in patients with typical Rett syndrome (*n* = 228): relationships between lung ventilation-perfusion (*V*/*Q*) inequalities patterns and respiratory variables.

Variables	Pulmonary ventilation/perfusion (*V*/*Q*) patterns in typical Rett syndrome					*P* value (ANOVA)
	No mismatch (*N* = 44)	“Low” (*N* = 64)	“High” (*N* = 73)	“Mixed” (*N* = 36)	“Simple” Mismatch (*N* = 11)	
*V*_tot_ (L/min)	6.74 ± 2.87^**a**^	5.26 ± 2.08^**a**,**b**^	9.71 ± 5.60^**b**,**c**,**d**^	6.07 ± 2.46^**c**^	6.3 ± 1.46^**d**^	**<0.001**
Respiratory rate (breaths/min)	27.4 ± 6.9	25.9 ± 9.1	30.4 ± 8.0	26.7 ± 8.3	27.1 ± 9.0	0.162
Alveolar vent. (L/min)	4.22 ± 2.60^**a**^	3.14 ± 1.80^**a**,**b**^	6.93 ± 4.80^**a**,**b**,**c**,**d**^	4.01 ± 2.2^**c**^	4.23 ± 1.30^**d**^	**<0.001**
PaO_2_ (mmHg)	95.4 ± 15.2^**a**,**b**^	85.7 ± 15.7^**a**,**d**^	92.2 ± 12.4^**c**,**d**^	83.9 ± 16.9^**b**,**c**^	89.8 ± 3.6	**0.025**
Std. PaO_2_ (mmHg)*	87.6 ± 14.4	81.2 ± 14.9	87.3 ± 11.2	78.7 ± 20.3	88.8 ± 6.5	0.082
PaCO_2_ (mmHg)	35.3 ± 8.5	36.3 ± 7.9	37.0 ± 6.6	36.9 ± 6.9	39.4 ± 3.7	0.753
Blood pH	7.429 ± 0.05	7.436 ± 0.05	7.417 ± 0.04	7.422 ± 0.04	7.413 ± 0.01	0.462
(A-a) O_2_ (mmHg)	14.1 ± 7.9^**a**,**b**,**c**,**d**,**e**^	25.1 ± 11.3^**a**,**b**,**d**^	27.8 ± 10.8^**a**,**c**,**d**^	36.9 ± 10.8^**a**,**b**,**c**,**d**,**e**^	19.4 ± 2.6^**b**,**c**,**d**,**e**^	**<0.001**
Bohr's DS % *V*_*t*_	45.0 ± 19.4^**a**,**c**,**d**^	41.2 ± 15.7^**b**,**c**,**d**^	55.4 ± 10.8^**c**^	54.7 ± 15.5^**a**,**b**,**d**^	41.8 ± 9.5^**a**,**c**,**d**^	**<0.001**
*Q*_*s*_/*Q*_*t*_ (%)	6.3 ± 2.9	24.3 ± 2.9	8.3 ± 7.2	19.4 ± 8.9	21.6 ± 15.1	0.224
*V*_O_2__ (mL/min)	137.8 ± 63.5^**a**^	89.5 ± 58.3^**a**,**b**,**c**,**d**^	181 ± 132^**b**,**c**,**d**^	88.6 ± 29.7^**d**,**e**^	164.8 ± 20.8^**e**^	**<0.001**
*V*_CO_2__ (mL/min)	148 ± 78	126 ± 58^**a**,**e**^	193 ± 146^**a**,**b**^	117 ± 55^**b**,**e**^	174 ± 59^**e**^	**0.015**
Respiratory ratio	1.12 ± 0.43^**a**^	1.72 ± 0.77^**a**,**b**,**c**,**d**^	1.09 ± 0.30^**b**^	1.37 ± 0.59^**c**^	1.04 ± 0.25^**d**^	**<0.001**

Data are expressed as means ± SD. Bold characters indicate statistical significant differences; superscript letters indicate significant pairwise post hoc differences; *V*_tot_: total ventilation; (A-a) O_2_: Bohr's DS: physiological dead space, as calculated by the Bohr equation, which, by quantifying the ratio of physiological dead space to the total tidal volume (*V*_*d*_/*V*_*t*_ = PaCO_2_ − PaCO_2_/PaCO_2_), gives an indication of the extent of wasted ventilation; (A-a) O_2_: O_2_ alveolar-arterial gradient; *V*_*t*_: tidal volume; *Q*_*s*_/*Q*_*t*_: pulmonary functional shunting; *V*_O_2__: oxygen uptake; *V*_CO_2__: carbon dioxide production; *values were calculated according to the formula by Sorbini et al. accounting for hypocapnia: standard PaO_2_ = 1.66 × PaCO_2_ + PaO_2_ − 66.4 [52]; respiratory ratio: respiratory exchange ratio, that is, *V*_CO_2__/*V*_O_2__. See text for further methodology details.

**Table 3:** Redox/antioxidant status in patients with typical Rett syndrome: systemic oxidative stress with decreased GSH/GSSG ratio.

Redox and antioxidant markers	Rett syndrome (*n* = 228)	Healthy controls (*n* = 114)	*P* value
P-NPBI (nmol/mL)	0.90 ± 0.18	0.43 ± 0.25	<0.0001
IE-NPBI (nmol/mL)	1.20 ± 0.30	0.78 ± 0.17	<0.0001
F_2_-IsoPs (pg/mL)	70.1 ± 20.5	24.2 ± 11.5	<0.0001
GSH (*µ*mol/L)	1673.0 ± 591.0	1165.0 ± 132.0	<0.0001
GSSG (*µ*mol/L)	179.0 ± 73.9	3.55 ± 1.90	<0.0001
GSH to GSSG ratio	10.9 ± 5.5	160.0 ± 61.0	<0.0001

P-NPBI: plasma non-protein-bound iron; IE-NPBI: intraerythrocyte non-protein-bound iron; F_2_-IsoPs: plasma F_2_-isoprostanes; GSH: reduced glutathione; GSSG: oxidized glutathione.

**Table 4:** Results of cardiorespiratory monitoring in patients with typical Rett syndrome (*n* = 228) confirming a high frequency of apneas and hypopneas either during wakefulness or sleep states.

Recorded events	Median events/h	Interquartile range
Sleep		
Obstructive apneas	6.2	3.4–58
Central apneas	0.4	0.15–0.92
Mixed apneas	1.5	0.4–2.5
Hypopneas	25.6	20.1–34.7

Wakefulness		
Obstructive apneas	17.7	4.9–11.38
Central apneas	0.7	0.08–1.07
Mixed apneas	1.7	0.92–2.4
Hypopneas	22	12.7–26

Apnoeas were defined as a >90% airflow decrease for ≥10 sec; hypopnoeas were defined as a >50% airflow reduction for ≥10 sec associated with a decrease of ≥3% in oxygen saturation [51]. Obstructive apneas refer to recorded events with cessation of airflow for ≥10 sec associated with persistent respiratory effort; central apneas refer to events characterized by cessation of airflow for ≥10 sec without associated respiratory effort; mixed apneas refer to respiratory events that begin as central apneas and end up as obstructive apneas.

**Table 5:** Correlation matrix between redox/antioxidant status and severity of recorded apnoeas, either during wakefulness or sleep, in patients with typical Rett syndrome (*N* = 228).

Redox and antioxidant markers	Apnoeas		
	Mild	Moderate	Severe
P-NPBI	0.185 (0.1431)	0.176 (0.1631)	**0.265** **(0.0346)**
IE-NPBI	**0.324** **(0.0089)**	**0.373** **(0.0024)**	**0.358** **(0.0037)**
F_2_-IsoPs	0.2220 (0.0800)	0.225 (0.0744)	**0.305** **(0.0142)**
GSH	**0.252** **(0.0449)**	0.101 (0.4262)	0.183 (0.1485)
GSSG	**0.258** **(0.0392)**	**0.260** **(0.0378)**	**0.267** **(0.033)**
GSH/GSSG ratio	−0.210 (0.0961)	**−0.247** **(0.0488)**	−0.241 (0.0552)

Data are expressed as rank correlation rho coefficients with *P* values in brackets. Bold characters indicate statistically significant associations. Apnoeas were defined as a >90% airflow decrease for ~10 sec; hypopneas were defined as a >50% airflow reduction for ≥10 sec associated with a decrease of ≥3% in oxygen saturation [50]. Apnoeas were further categorized as mild (10 to 15 sec), moderate (15 to 30 sec), and severe (>30 sec) on the basis of their recorded duration. Legends: P-NPBI: plasma non-protein-bound iron; IE-NPBI: intraerythrocyte non-protein-bound iron; F_2_-IsoPs: plasma F_2_-isoprostanes; GSH: reduced glutathione; GSSG: oxidized glutathione.

**Table 6:** Frequency/severity of apneas, recorded during either the wakefulness or sleep, identifies Rett patients with increased intraerythrocyte non-protein-bound iron (IE-NPBI) levels: receiver operating characteristic (ROC) curves analyses.

Variable	AUC ± SE	95% C.I.	*P*-value	Criterion	Sens.%	Spec.%	+LR	−LR	+PV	−PV
Total apneas/h	0.690 ± 0.0669	**0.563–0.799**	**0.0044**	**>7.4**	**53.8**	**82.05**	**1.5**	**0.89**	**56.2**	**62.7**
Mild apneas/h	0.634 ± 0.0714	0.504–0.751	0.0605	>1	30.7	89.5	1.54	0.51	51.4	74.1
Moderate apneas/h	0.664 ± 0.0687	**0.563–0.778**	**0.0163**	**>0.8**	**80.7**	**55.3**	**1.81**	**0.35**	**55.3**	**80.8**
Severe apneas/h	0.670 ± 0.0693	**0.541–0.782**	**0.0142**	**>2**	**50**	**81.6**	**2.71**	**0.61**	**65**	**70.5**

AUC: area under the curve; SE: standard error; Sens.: sensitivity; Spec: specificity; +LR: positive likelihood ratio; −LR: negative likelihood ratio; +PV: positive predictive value; −PV: negative predictive value. Bold characters indicate statistically significant items.

**Table 7:** Relationships between lung ventilation/perfusion (*V*/*Q*) patterns and the redox/antioxidant status in patients with typical Rett syndrome (*n* = 228).

Redox and antioxidant markers	Pulmonary ventilation/perfusion (*V*/*Q*) patterns in typical Rett syndrome					*P* value (ANOVA)
	No mismatch (*N* = 44)	“Low” (*N* = 64)	“High” (*N* = 73)	“Mixed” (*N* = 36)	“Simple” mismatch (*N* = 11)	
P-NPBI (nmol/mL)	0.50 ± 0.32^**a**^	0.86 ± 0.07^**a**,**b**,**c**^	0.91 ± 0.15^**a**^	1.02 ± 0.22^**a**,**b**,**c**^	0.71 ± 0.05^**b**,**c**^	**<0.001**
IE-NPBI (nmol/mL)	0.80 ± 0.24^**a**^	1.04 ± 0.05^**a**,**b**^	1.20 ± 0.21^**a**,**b**^	1.30 ± 0.49^**a**,**b**^	0.96 ± 0.13^**b**^	**<0.001**
F_2_-IsoPs (pg/mL)	27.3 ± 11.1^**a**^	65.2 ± 14.4^**a**,**b**^	76.5 ± 13.2^**a**,**b**^	100.8 ± 11.4^**a**,**b**^	46.2 ± 7.9^**b**^	**<0.001**
GSH (*μ*mol/L )	1206 ± 140^**a**^	1867 ± 759^**a**,**b**^	1794 ± 507^**a**,**b**^	1442 ± 373^**a**,**b**^	1419 ± 523^**b**^	**<0.001**
GSSG (*μ*mol/L )	8.0 ± 3.4^**a**^	193.6 ± 85.3^**a**,**b**^	222.5 ± 61.5^**a**,**b**^	132.0 ± 25.9^**a**^	144.3 ± 71.4^**b**^	**<0.001**
GSH/GSSG ratio	175 ± 83^**a**^	12.2 ± 7.8^**a**^	8.2 ± 1.9^**a**^	11.6 ± 5.1^**a**^	11.1 ± 5.2^**a**^	**<0.001**

Data are expressed as means ± SD. Bold characters indicate statistical significant differences; superscript letters indicate significant pairwise post hoc differences; P-NPBI: plasma non-protein-bound iron; IE-NPBI: intraerythrocyte non-protein-bound iron; F_2_-IsoPs: plasma F_2_-isoprostanes; GSH: reduced glutathione; GSSG: oxidized glutathione.

## Abbreviations

(A-a) O_2_: O_2_ alveolar-arterial pulmonary gradient
AHI: Apnea-hypopnea index
ASDs: Autism Spectrum Disorders
AUC: Area under the curve
Bohr's DS: Physiological dead space as calculated by the Bohr equation
*CDKL5*: Cyclin-dependent kinase-like 5
F_2_-IsoPs: F_2_-isoprostanes
*FOXG1*: Forkhead box G1
GEA: Lung gas exchange abnormality
GSH: Reduced glutathione
GSSG: Oxidized glutathione
IE-NPBI: Intraerythrocyte non-protein-bound iron
MeCP2: Methyl-CpG-binding protein 2
OS: Oxidative stress
OSAHS: Obstructive sleep apnea-hypopnea syndrome
PaCO_2_: Partial arterial pressure of CO_2_
PaO_2_: Partial arterial pressure of O_2_
P-NPBI: Plasma non-protein-bound iron
RB-ILD: Respiratory bronchiolitis-associated interstitial lung disease
*Q*_*s*_/*Q*_*t*_: Pulmonary functional shunting (i.e., venous admixture)
RTT: Rett syndrome
*V*/*Q*: Lung ventilation-to-perfusion ratio
*V*_*t*_: Tidal volume
*V*_tot_: Total ventilation
*V*_CO_2__: CO_2_ production
*V*_O_2__: O_2_ uptake.
